# Glycemic Control Status After Six Months in Post-COVID-19 Patients

**DOI:** 10.7759/cureus.81225

**Published:** 2025-03-26

**Authors:** Chowdhury Adnan Sami, Mohammad Ferdous Ur Rahaman, Md Mizanur Rahman Khan, Mohammad Monzurul Alam Bhuiyan, Md. Abdul Matin, Hasan M Rashed, Abed H Khan, Shohael Mahmud Arafat, Md. Nazmul Hasan

**Affiliations:** 1 Internal Medicine, Bangabandhu Sheikh Mujib Medical University, Dhaka, BGD; 2 Laboratory Medicine, Bangabandhu Sheikh Mujib Medical University, Dhaka, BGD

**Keywords:** covid-19, diabetes mellitus, glycemic control, hyperglycemia, long covid-19, new-onset diabetes, post-covid-19 sequelae

## Abstract

Background

Despite being typically a viral respiratory disease, COVID-19 has harmful effects that go beyond the respiratory system. The endocrine system is particularly susceptible to damage due to the high expression of angiotensin-converting enzyme-2 receptors. This study evaluates glycemic status in survivors of COVID-19.

Methodology

In this prospective, observational study, 96 individuals were enrolled from the COVID-19 unit of Bangabandhu Sheikh Mujib Medical University (BSMMU). Mild and moderate COVID-19 patients were classified as non-severe, whereas severe and critical cases were classified as severe, following the WHO disease severity classification. Follow-ups were conducted at the post-COVID-19 clinic at BSMMU one and six months after diagnosis. Blood samples for fasting blood sugar and glycated hemoglobin measurements were collected within 24 hours of initial diagnosis and during each follow-up at the first and sixth months.

Results

Of the 96 participants, the non-severe and severe groups consisted of 49 (51%) and 47 (49%) participants, respectively. Among the participants, 62 (63.9%) were men, the mean age was 54.2 (15.9) years, and hypertension was the most common comorbidity (37, 38.5%). After six months, 12 new cases of diabetes (15.4%) were observed, with a male predominance (10, 62%). After adjusting for potential confounders, we found that severe COVID-19 was substantially linked to a higher risk of diabetes at six months (odds ratio = 5.5, 95% confidence interval, 1.1-27.7, p = 0.03).

Conclusions

The study findings showed a significant association between a higher frequency of diabetes and severe COVID-19.

## Introduction

A COVID-19 infection has far more health risks than its initial infection of the respiratory system. Long-term sequelae have been seen with its predecessor, severe acute respiratory syndrome (SARS-CoV-1), and are now also documented in the case of SARS-CoV-2 [1,2]. The association between COVID-19 and acute new-onset hyperglycemia has been reported in the literature. Among COVID-19 patients who previously did not have diabetes, concerns of irreversible beta-cell damage after a single episode of the infection were heightened [3]. Given the high level of angiotensin-converting enzyme-2 (ACE2) expression in the pancreatic islets and prior experiences with new-onset diabetes in SARS-CoV-1 infection, there is a substantial concern regarding the relationship between hyperglycemia and COVID-19 [4].

After attaching itself to the target host cell’s ACE2 receptor, SARS-CoV-2 internalizes and multiplies [5]. Lazartigues et al. supported the idea that the endocrine system is involved in viral infection by confirming the expression of ACE2 and transmembrane protease serine 2 mRNA in endocrine tissue in both males and females [6]. ACE2 is expressed in the pancreas, causing increased mRNA levels in the exocrine pancreas and the islets than in the lungs, resulting in destruction [7]. Inflammatory cytokines such as interleukin 6 are released after the destruction of pancreatic islet cells, as well as activate initiating autoimmunity in genetically susceptible patients [8,9].

The long-term consequences of COVID-19 on hyperglycemia are still unclear. A study indicated that 63% of patients with hyperglycemia at admission had returned to a euglycemic status six months later [10]. Similarly, 5% of individuals who were admitted with new-onset diabetes still had diabetes three years after SARS-CoV-1 infection [4].

This study aims to evaluate the hyperglycemic status of COVID-19 patients, identify newly diagnosed diabetes in the post-COVID-19 cohort, and assess glycemic control in patients who were already diabetic to ascertain whether these abnormalities are temporary or permanent, even though the long-term effects of COVID-19 on hyperglycemia are still unknown.

## Materials and methods

This prospective, longitudinal study was conducted in the Department of Internal Medicine at Bangabandhu Sheikh Mujib Medical University (BSMMU) from October 2020 to September 2022. Patients were recruited from triage and the COVID-19 unit and then followed up at the post-COVID-19 clinic.

Study population

Hospital-admitted adult patients who had positive reverse transcription polymerase chain reaction (RT-PCR) for COVID-19 were recruited for this study. Patients who were taking oral or intravenous steroids before hospital admission, those taking known medications, or those known to have conditions causing an increase in cortisol-binding globulin (CBG) (oral contraceptive pills, known pregnancy, known end-stage renal disease); patients with a condition known to decrease the level of CBG (cirrhosis, nephrotic syndrome, hyperthyroidism); and patients with previously documented adrenal insufficiency were excluded from the study. We recruited patients with previous diabetes in both the severe and non-severe groups. This diabetic cohort was also analyzed separately from the non-diabetic cohort while analyzing the new-onset diabetic status. After applying the inclusion and exclusion criteria, those who gave consent were recruited from triage or the COVID-19 unit at BSMMU. These recruited patients were further contacted by telephone to attend a follow-up visitation at the post-COVID-19 outpatient clinic at BSMMU, in person, at the first month and sixth month following the diagnosis of COVID-19.

Data collection

From the eligible patients, a proper history was taken, and a physical examination was performed. Data during the COVID-19 episode were collected from the COVID-19 triage or the COVID-19 unit. Each participant went through two follow-ups, scheduled at one and six months from the date of a positive RT-PCR test for COVID-19. All participants were divided into two groups, namely, the non-severe and severe groups, according to the WHO disease severity. The non-severe group included patients with mild and moderate disease severity, and the severe and critical patients were included in the severe group. Blood samples (5 mL) were collected by venipuncture into plain tubes for fasting blood sugar (FBS) and glycated hemoglobin (HbA1c) within 24 hours of admission. FBS and HbA1c measurements were performed by the automated analyzer (Alinity CI, Sebia, Lisses, France) in the Department of Biochemistry Laboratory.

Statistical analysis

SPSS version 28.0 for Windows (IBM Corp., Armonk, NY, USA) was used to analyze the data. Comorbidities, sex, and other qualitative factors were represented as percentages. The mean ± standard deviation was used to express the quantitative data, such as age. When applicable, the chi-square test was used to analyze qualitative data. The relationship between continuous variables was assessed using an independent t-test. To determine whether a quantitative variable changed significantly over time, a repeated-measures two-way analysis of variance test was used. The 95% confidence interval (CI) and odds ratios (OR) for the associations were estimated using multivariable adjusted logistic regression models. The threshold for statistical significance was set at p-values <0.05.

Ethical considerations

Participants were recruited after obtaining ethical approval from the Institutional Review Board of BSMMU (approval number: BSMMU/2021/4699). All participants were informed in detail about the nature and purpose of the study. Informed written consent was taken from all patients before enrolment.

## Results

Baseline characteristics

Table 1 displays the participants’ clinical and demographic attributes. There were 47 (49%) participants in the severe group and 49 (51%) in the non-severe group. Of the 96 individuals who were available for the six-month follow-up, 34 (35.1%) were women and 62 (63.9%) were men. Participants were 54.2 (15.9) years old on average, while severe patients were older at 62 years (p < 0.01). The average body mass index (BMI) of the participants was 23.2 (3.2) kg/m^2^. A smoking history was present in 29 (30.2%) patients. Hypertension (37, 38.5%), diabetes (18, 18.7%), cardiovascular disease (12, 12.5%), asthma (14, 14.6%), chronic obstructive pulmonary disease (COPD) (19, 19.8%), chronic renal disease (14, 14.6%), and chronic liver disease (3, 3.1%) were the most prevalent comorbidities. The severe group had increased rates of COPD, diabetes, and hypertension, although not statistically significant. Dexamethasone was administered to all patients in the severe group and four (8.4%) patients in the non-severe group (Table 1).

**Table 1:** Sociodemographic characteristics of COVID-19 patients. *: Independent t-tests were done; **: chi-square test was done. NIV: non-invasive ventilation; COPD: chronic obstructive pulmonary disease; BMI: body mass index

Characteristics	Total cohort, n = 96	Non-severe, n = 49	Severe, n = 47	Chi square/t-test value	P-value
Age (years), mean (SD)	54.2 (15.9)	46.1 (15.4)	62 (11.2)	5.27	<0.01*
Gender					
Male	62 (63.9%)	31 (63.3%)	31 (66%)		
Female	34 (35.1%)	18 (36.7%)	16 (34%)		
BMI	23.2 (3.2)	22.7 (2.8)	24.1 (4.1)		
Obese	36 (37.5%)	16 (32.7%)	20 (42.6%)	1.21	0.317**
Smoker	29 (30.2%)	14 (28.5%)	15 (31.9%)	0.96	0.916**
Non Smoker	49 (51%)	26 (53.1%)	23 (48.9%)		
Ex-smoker	18 (18.8%)	9 (18.4%)	9 (19.1%)		
Comorbidities					
Diabetes	18 (18.7%)	7 (14.2%)	11 (23.4%)	1.57	0.25**
Hypertension	37 (38.5%)	14 (28.6%)	23 (48.1%)	2.87	0.04**
Cardiovascular disease	12 (12.5%)	4 (8.2%)	8 (17%)	1.81	0.19**
Asthma	14 (14.6%)	7 (14.3%)	7 (14.9%)	0.89	0.93**
COPD	19 (19.8%)	4 (8.2%)	15 (31.9%)	4.15	0.004**
Chronic kidney disease	14 (14.6%)	5 (10.2%)	9 (19.1%)	1.61	0.21**
Chronic liver disease	3 (3.1%)	1 (2%)	2 (4.3%)	1.07	0.53**
Use of drugs					
Dexamethasone	51 (53.1%)	4 (8.2%)	47 (100%)	5.87	<0.001**
Remdesivir	45 (46.8%)	-	45 (95.6%)		
Tocilizumab	12 (12.5%)	-	12 (25.5%)		
Baricitinib	8 (8.3%)	-	8 (17%)		
Ventilation					
NIV	16 (16.7%)	-	16 (34%)		
Intubation	4(4.2%)	-	4 (8.5%)		

Glycemic control in post-COVID-19 patients

**Figure 1:**
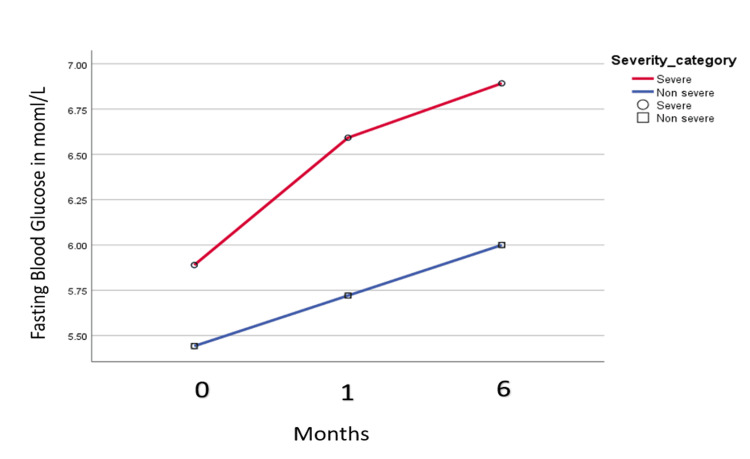
Fasting blood sugar level in the severe and non-severe groups in the hospital and first and six months after discharge.

The mean FBS level increased from baseline (during COVID-19) to the first month and six months post-discharge. In the non-severe group, the mean FBS (mmol/L) in the hospital was 5.4 (0.7), after one month was 5.6 (1.0), and after six months was 5.9 (0.9) post-discharge. In the severe case group, the mean FBS (mmol/L) was 5.8 (1.2), 6.4 (1.2), and 6.9 (1.1) in the hospital, one month, and six months post-discharge (Figure 1).

**Figure 2:**
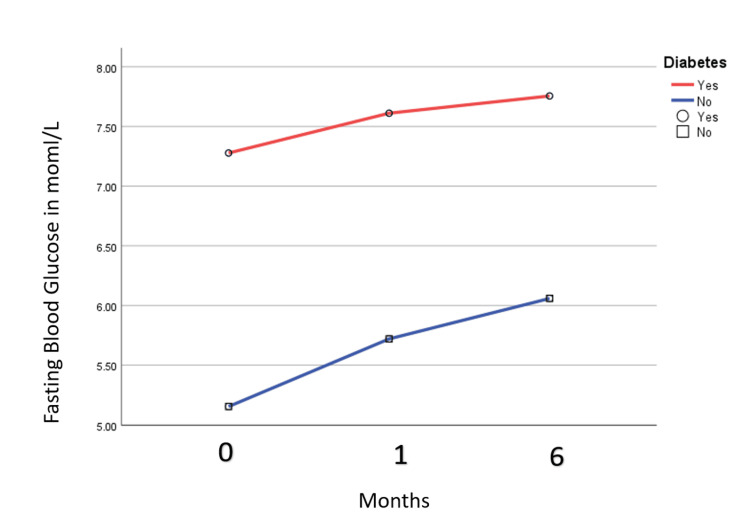
Fasting blood sugar level in the previously diabetic and non-diabetic group in the hospital and first and six months after discharge.

In the known diabetic cohort, a similar trend of rising blood sugar was noted. The mean FBS (mmol/L) level increased from baseline (in hospital) to the first month and six months post-discharge. In the known diabetic cohort, the mean FBS (mmol/L) in the hospital was 7.3 (0.5), at one month after discharge was 7.6 (0.6), and at six months after discharge was 7.7 (0.8). In the non-diabetic group, the mean FBS (mmol/L) was 5.1 (0.6), 5.6 (0.9), and 6.01 (0.8) in the hospital, one month, and six months post-discharge (Figure 2).

**Figure 3:**
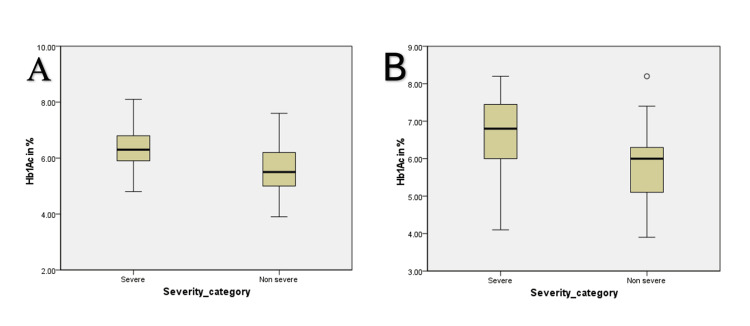
Glycated hemoglobin level after the first month (A) and six months (B) of discharge in severe and non-severe group

HbA1c trend was also seen to be steadily rising. We did not assess baseline HbA1c, as it reflects the prior three months’ glycemic control status; hence, we only assessed the first and sixth month HbA1c status. In the non-severe group, the mean HbA1c was 5.6% (0.7) in the first month and 5.8% (0.9) in the sixth month (p = 0.001). In the case of the severe group, the mean HbA1c was 6.2% (0.7) in the first month and 6.6% (1.0) in the sixth month (p = 0.001) (Figure 3, Table 2).

**Table 2:** Changes in the FBS and HbA1c level after the first and six months of discharge in the severe and non-severe group. *: Independent t-test was performed. CI: confidence interval; FBS: fasting blood sugar; HbA1c: glycated hemoglobin

Glycemic status, mean (SD)	Non-severe, mean (SD)	Severe, mean (SD)	t-test value	P-value*	95% CI
FBS, first month (mmol/L)	5.6 (1.0)	6.4 (1.2)	2.54	0.002	0.29–1.2
FBS, sixth month (mmol/L)	5.9 (0.9)	6.9 (1.1)	3.32	0.001	0.48–1.9
Hb1Ac, first month	5.6 (0.7)	6.2 (0.73)	4.21	0.001	0.38–0.98
Hb1Ac, sixth month	5.8 (0.9)	6.6 (1.0)	3.37	0.001	0.35–1.2

Risk factor analysis

There were 12 occurrences of diabetes (15.4%) observed six months after discharge. Among them, males were predominant at 10 (62%). Severe COVID-19 was associated with a higher frequency of new-onset diabetes after six months from the initial diagnosis (OR = 5.5, 95% CI = 1.1 to 27.7, p = 0.03) (Table 3).

**Table 3:** Association of new-onset diabetes with different parameters six months post-discharge. *: Multivariable logistic regression analysis was done. CI: confidence interval

Parameter	Odds ratio	95% CI		P-value*
		Lower bound	Upper bound	
Severe COVID-19	5.5	1.1	17.7	0.03
Male	1.23	0.31	4.8	0.76
Hypertension	0.81	0.81	3.59	0.78
Chronic lung disease	0.9	0.15	6.31	0.97
Dexamethasone use	3.75	1.23	11.8	0.08
Overweight	1.37	0.95	1.87	0.15
Cardiovascular disease	0.52	0.5	5.1	0.57

## Discussion

This longitudinal study evaluated the glycemic state of patients six months after they were released from the COVID-19 unit of BSMMU. The final patient enrolment was done in October 2021, having begun in February 2021. Of the 96 patients we enrolled, 47 were in the severe group (severe and critical), and 49 were in the non-severe group (mild and moderate). Six months after discharge, we discovered that a higher risk of newer diabetes onset was linked to the severity of the condition, which occurred more in males.

Overall, 31 (66%) of the 47 individuals in the severe group were men. Sex differences in our study may be partially supported by current findings showing men are more likely than women to contract COVID-19, be hospitalized, have a more severe illness, have more intensive care unit admissions, and die from COVID-19 [11]. Furthermore, sex differences in the immunological response and genetic/epigenetic variables [12,13] and androgen-mediated ACE2 expression [14,15] may be reasons for sex-based differences. Additionally, we found that the severity of COVID-19 increased with age. The mean age of the severe group was 62 (11.2) years compared to 46.1 (15.4) years of the non-severe group. Comprehensive meta-analyses provide strong evidence for this severity connection with advancing age and male gender [16].

This study assessed the six-month glycemic status in the post-COVID-19 cohort. Multivariable logistic analysis revealed a significant correlation between severe COVID-19 and 12 (15.4%) cases of new-onset diabetes (OR = 5.5). Additionally, diabetes patients, particularly men, were more susceptible to managing their glycemic state. According to a previous study, Diabetes and COVID-19 have a reciprocal association [17]. A meta-analysis showed similar results to our findings, which involved eight studies and over 3,700 patients. According to the study, 14.4% of newly diagnosed diabetes was noted in hospitalized post-acute COVID-19 patients [18], which was similar to this study. However, in a different study of a post-COVID-19 cohort, where follow-up was done for more than one year, new-onset diabetes was found in 8.6% of the participants [19], which was a lower rate of diabetes incidence than this study. One possible explanation could be that the study showed diabetes incidence 12 months after COVID-19, whereas we showed follow-up at six months. A previous study of the SARS virus (2003) found initial hyperglycemia in 51% (20/39) of the participants, but tended to reduce three years following SARS. Only 5% of patients remained diabetic three years following the initial diagnosis [4]. Therefore, longer follow-up may show if hyperglycemia in this cohort declines or persists. In a retrospective study, Barrett et al. demonstrated that individuals with COVID-19 are diagnosed with diabetes at a higher rate than those without the virus and those in the prepandemic period [20]. A retrospective study from Germany showed that COVID-19 increases the incidence of type 2 diabetes from matched controls, which corroborated our findings [21]. Although the exact mechanisms are still unknown, current evidence suggests that COVID-19-related diabetes may be caused by the virus’s direct impact on beta-cells of the pancreas via ACE2 receptors [22,23] and immune dysregulation [24,25]. According to other recent studies, COVID-19 may raise blood sugar levels temporarily and have long-term effects that raise the chance of developing diabetes in the future [25,26]. Previous studies demonstrated that dexamethasone can cause hyperglycemia, but it was temporary. In most studies, it has been hypothesized that dexamethasone is one of the factors of dysregulated hyperglycemia along with exacerbated proinflammatory response and beta-cell dysfunction [27].

However, the findings of this study also suggested that the stress response and glucocorticoid therapy might not be the only factors contributing to the new diabetes event. Although to validate these findings, large prospective studies with longer follow-ups are necessary, which would improve the internal validity and causal inference of associations between COVID-19 and new-onset diabetes.

There are various limitations to our investigation. There was no control group to project severe COVID-19 as a risk factor alone. The study was unable to identify any risk variables for incident diabetes or post-sequelae of diabetes. The results might not be as generalizable as they could be because of the single-city setting, single-center design, and small sample size. However, the results would have been improved with diverse groups from various regions throughout the country. There could be a possibility of recall bias in self-reporting data on family history and lifestyles, which may affect the exposure assessment in this study. Type 1 and type 2 diabetes were not differentiated. However, considering the age of the participants (>50 years old), we hypothesized that type 2 diabetes would account for the majority of post-infection diabetes in our study. Moreover, the confidence interval is too broad, which might decrease the strength of the study.

## Conclusions

In post-COVID-19 patients, our prospective, observational analysis revealed a strong correlation between severe COVID-19 and a higher likelihood of developing new-onset diabetes, which is more common in men. Concerns over the long-term metabolic effects of SARS-CoV-2 were heightened as the severity of COVID-19 is a powerful predictor of post-infection glycemic dysregulation. These results highlight the necessity of ongoing glycemic monitoring in post-COVID-19 patients, especially those who had severe disease, to provide early identification and treatment of newly diagnosed diabetes. To validate our findings and investigate the underlying processes that connect the severity of COVID-19 to metabolic disruptions, more extensive, multicenter studies with longer follow-up times are needed.

## References

[1] Nasserie T, Hittle M, Goodman SN (2021). Assessment of the frequency and variety of persistent symptoms among patients with COVID-19: a systematic review. JAMA Netw Open.

[2] Løkke FB, Hansen KS, Dalgaard LS, Öbrink-Hansen K, Schiøttz-Christensen B, Leth S (2023). Long-term complications after infection with SARS-CoV-1, influenza and MERS-CoV - lessons to learn in long COVID?. Infect Dis Now.

[3] Chee YJ, Ng SJ, Yeoh E (2020). Diabetic ketoacidosis precipitated by Covid-19 in a patient with newly diagnosed diabetes mellitus. Diabetes Res Clin Pract.

[4] Yang JK, Lin SS, Ji XJ, Guo LM (2010). Binding of SARS coronavirus to its receptor damages islets and causes acute diabetes. Acta Diabetol.

[5] Chen Y, Guo Y, Pan Y, Zhao ZJ (2020). Structure analysis of the receptor binding of 2019-nCoV. Biochem Biophys Res Commun.

[6] Lazartigues E, Qadir MM, Mauvais-Jarvis F (2020). Endocrine significance of SARS-CoV-2's reliance on ACE2. Endocrinology.

[7] Liu F, Long X, Zhang B, Zhang W, Chen X, Zhang Z (2020). ACE2 expression in pancreas may cause pancreatic damage after SARS-CoV-2 infection. Clin Gastroenterol Hepatol.

[8] Roy S, Pokharel P, Piganelli JD (2024). Decoding the immune dance: unraveling the interplay between beta cells and type 1 diabetes. Mol Metab.

[9] Hirano T (2021). IL-6 in inflammation, autoimmunity and cancer. Int Immunol.

[10] Montefusco L, Ben Nasr M, D'Addio F (2021). Acute and long-term disruption of glycometabolic control after SARS-CoV-2 infection. Nat Metab.

[11] Pijls BG, Jolani S, Atherley A (2022). Temporal trends of sex differences for COVID-19 infection, hospitalisation, severe disease, intensive care unit (ICU) admission and death: a meta-analysis of 229 studies covering over 10M patients. F1000Res.

[12] Wilkinson NM, Chen HC, Lechner MG, Su MA (2022). Sex differences in immunity. Annu Rev Immunol.

[13] Takahashi T, Ellingson MK, Wong P (2020). Sex differences in immune responses that underlie COVID-19 disease outcomes. Nature.

[14] La Vignera S, Cannarella R, Condorelli RA, Torre F, Aversa A, Calogero AE (2020). Sex-specific SARS-CoV-2 mortality: among hormone-modulated ACE2 expression, risk of venous thromboembolism and hypovitaminosis D. Int J Mol Sci.

[15] Mohamed MS, Moulin TC, Schiöth HB (2021). Sex differences in COVID-19: the role of androgens in disease severity and progression. Endocrine.

[16] Peckham H, de Gruijter NM, Raine C (2020). Male sex identified by global COVID-19 meta-analysis as a risk factor for death and ITU admission. Nat Commun.

[17] Rubino F, Amiel SA, Zimmet P (2020). New-onset diabetes in Covid-19. N Engl J Med.

[18] Sathish T, Kapoor N, Cao Y, Tapp RJ, Zimmet P (2021). Proportion of newly diagnosed diabetes in COVID-19 patients: a systematic review and meta-analysis. Diabetes Obes Metab.

[19] Zhang J, Shu T, Zhu R, Yang F, Zhang B, Lai X (2022). The long-term effect of COVID-19 disease severity on risk of diabetes incidence and the near 1-year follow-up outcomes among postdischarge patients in Wuhan. J Clin Med.

[20] Barrett CE, Koyama AK, Alvarez P (2022). Risk for newly diagnosed diabetes >30 days after SARS-CoV-2 infection among persons aged <18 years - United States, March 1, 2020-June 28, 2021. MMWR Morb Mortal Wkly Rep.

[21] Rathmann W, Kuss O, Kostev K (2022). Incidence of newly diagnosed diabetes after Covid-19. Diabetologia.

[22] Yang L, Han Y, Nilsson-Payant BE (2020). A human pluripotent stem cell-based platform to study SARS-CoV-2 tropism and model virus infection in human cells and organoids. Cell Stem Cell.

[23] Sathish T, Tapp RJ, Cooper ME, Zimmet P (2021). Potential metabolic and inflammatory pathways between COVID-19 and new-onset diabetes. Diabetes Metab.

[24] Lazzaroni MG, Piantoni S, Masneri S (2021). Coagulation dysfunction in COVID-19: the interplay between inflammation, viral infection and the coagulation system. Blood Rev.

[25] Landstra CP, de Koning EJ (2021). COVID-19 and diabetes: understanding the interrelationship and risks for a severe course. Front Endocrinol (Lausanne).

[26] Wu L, Girgis CM, Cheung NW (2020). COVID-19 and diabetes: insulin requirements parallel illness severity in critically unwell patients. Clin Endocrinol (Oxf).

[27] Rhou YJ, Hor A, Wang M, Wu YF, Jose S, Chipps DR, Cheung NW (2022). Dexamethasone-induced hyperglycaemia in COVID-19: glycaemic profile in patients without diabetes and factors associated with hyperglycaemia. Diabetes Res Clin Pract.

